# Centredness in health care: A systematic overview of reviews

**DOI:** 10.1111/hex.13461

**Published:** 2022-03-08

**Authors:** Caroline Feldthusen, Emma Forsgren, Sara Wallström, Viktor Andersson, Noah Löfqvist, Richard Sawatzky, Joakim Öhlén, Eva J. Ung

**Affiliations:** ^1^ Institute of Health and Care Sciences, Sahlgrenska Academy University of Gothenburg Gothenburg Sweden; ^2^ University of Gothenburg Centre for Person‐Centred Care (GPCC) University of Gothenburg Gothenburg Sweden; ^3^ School of Nursing Trinity Western University Langley British Columbia Canada; ^4^ Centre for Health Evaluation and Outcome Sciences, Providence Health Care Vancouver British Columbia Canada; ^5^ Palliative Centre, Sahlgrenska University Hospital, Region Västra Götaland Gothenburg Sweden

**Keywords:** family‐centred care, overview of reviews, patient‐centred care, person‐centred care, person‐centredness

## Abstract

**Introduction:**

The introduction of effective, evidence‐based approaches to centredness in health care is hindered by the fact that research results are not easily accessible. This is partly due to the large volume of publications available and because the field is closely linked to and in some ways encompasses adjoining fields of research, for example, shared decision making and narrative medicine. In an attempt to survey the field of centredness in health care, a systematic overview of reviews was conducted with the purpose of illuminating how centredness in health care is presented in current reviews.

**Methods:**

Searches for relevant reviews were conducted in the databases PubMed, Scopus, Cinahl, PsychINFO, Web of Science and EMBASE using terms connected to centredness in health care. Filters specific to review studies of all types and for inclusion of only English language results as well as a time frame of January 2017–December 2018, were applied.

**Results:**

The search strategy identified 3697 unique reviews, of which 31 were included in the study. The synthesis of the results from the 31 reviews identified three interrelated main themes: Attributes of centredness (what centredness is), Translation from theory into practice (how centredness is done) and Evaluation of effects (possible ways of measuring effects of centredness). Three main attributes of centeredness found were: being unique, being heard and shared responsibility. Aspects involved in translating theory into practice were sufficient prerequisites, strategies for action and tools used in safeguarding practice. Further, a variety and breadth of measures of effects were found in the included reviews.

**Conclusions:**

Our synthesis demonstrates that current synthesized research literature on centredness in health care is broad, as it focuses both on explorations of the conceptual basis and the practice, as well as measures of effects. This study provides an understanding of the commonalities identified in the reviews on centredness in healthcare overall, ranging from theory to practice and from practice to evaluation.

**Patient or Public Contribution:**

Patient representatives were involved during the initiation of the project and in decisions about its focus, although no patient or public representatives made direct contributions to the review process.

## 1 INTRODUCTION

Care that embraces the values and preferences of patients and is achieved in an alliance or partnership between the patients and professionals is desired by patients and relatives (https://www.whatmatterstoyou.scot/) as well as promoted by the World Health Organization.^1^, ^2^ This way of managing care is often described in terms of centredness (person‐centredness, patient‐centredness, family‐centredness) and centred care. There is also evidence showing that person‐centred care can improve the quality of care and reduce costs in particular settings.^3^, ^4^, ^5^, ^6^, ^7^ As a result, healthcare authorities have made decisions towards implementing person‐centred care in several countries, including the United Kingdom,^8^ Canada,^9^ United States^10^ and Sweden.^11^

Effective evidence‐based introduction of centredness in health care is nevertheless hindered by the fact that research results are not easily accessible. These challenges are in part due to a large number of publications available and challenges in delimitation. Centredness in health care relates to and incorporates adjoining fields of research, such as shared decision‐making and narrative medicine. In addition, only one Medical Subject Heading (MeSH)—patient‐centred care—exists at present and despite this, it is not widely used. Moreover, and more importantly, this MeSH does not correspond to the conceptual description of, say, ‘person‐centred care’ or ‘family‐centred care’, and thus does not capture the breadth of centredness in health care. Another challenge is the lack of conceptual and terminological clarity in the field. Attempts have been made to differentiate between constructs, suggesting, for example, that the goal of care is different when comparing person‐ and patient‐centred care. Another difference may be in the underlying theories and philosophies by which conceptualizations of centredness are constructed.^12^ However, others state that the main difference in terminology depends on the context and patient group in focus, and that conceptual differences between constructs are minor.^13^

Centredness in health care is an evolving field that has expanded largely over a short period of time. Due to the large volume of literature available in the field of centredness in terms of both original research papers and systematic reviews, overviews of reviews have been compiled. This has been done to tackle conceptual confusion^12^ and as a means towards developing practice guidelines for professionals.^14^ To our knowledge, there are no overviews of reviews available, which explore multiple rather than single aspects of centredness, such as the conceptual foundation or professional practice. The aim of this systematic overview of reviews, therefore, is to illuminate how centredness in health care is portrayed in current reviews to develop a coherent overview of commonalities across concepts of centredness in health care.

## 2 MATERIALS AND METHODS

We decided to undertake a systematic overview of reviews of the field of centredness in health care, which entails synthesizing evidence from a collection of reviews.^15^, ^16^ Due to a large number of systematic reviews, we limited our overview to a 2‐year period. The data collection commenced in winter 2019 and therefore the two previous years were chosen (2017–2018).

### 2.1 Eligibility criteria

To be included in the current study, citations needed to explore, discuss or elaborate on centredness in health care (i.e., not simply include a term such as person‐centred care or person‐centredness). For the purpose of this review, a broad and inclusive definition of centredness was used: (I) care in which the patient's will, needs and desires are elicited and acknowledged and (II) working in a collaborative partnership involving patient, healthcare professionals and other people of importance in the patient's life. This definition is framed by the University of Gothenburg Centre for person‐centred care and the ethics described in a position paper by Ekman et al.^17^ Citations were included if they were reviews of empirical healthcare studies with systematic searches but not necessarily including quality assessment, published between January 2017 and December 2018, and written in English. Review protocols and reviews not including a description of a systematic search, screening and analysis were excluded. Citations with the main focus on selected aspects of centredness, such as shared decision‐making and narrative medicine, but not addressing the broader scope of centredness as defined above, were also excluded. Furthermore, citations from the contexts of criminal care, social services and general pedagogics/education were excluded.

### 2.2 Information sources

**Figure 1:**
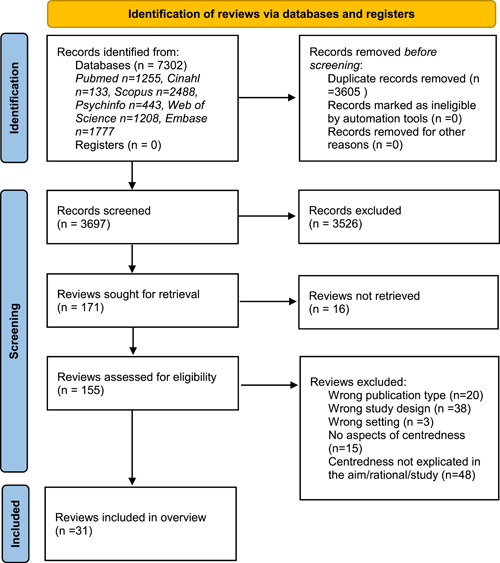
Flowchart demonstrating the selection process of included reviews

The literature search strategy was developed by the team in collaboration with two experienced medical librarians with expertize in systematic review searching. Searches were then conducted in the databases PubMed, Scopus, Cinahl, PsychINFO, Web of Science and EMBASE and adapted to the specifications of each database. The final search strategy in PubMed can be found in Appendix A. No follow‐up searches were conducted, as the period of this overview of reviews was limited to a specific time frame. The final search results were exported into the software EndNote and duplicates were removed (Figure 1).

### 2.3 Selection of sources of evidence

The search strategy identified 3697 reviews after the removal of duplicates. Titles and abstracts were read for all 3697 reviews, of which 171 reviews were further selected for full‐text reading.

Screening of titles and abstracts was conducted in the online application Rayyan.^18^ Five assessors (C. F., E. F., S. W., J. Ö., E. J. U.) working in pairs (one assessor participated in two pairs) evaluated the titles and abstracts against eligibility criteria. Disagreements were resolved within pairs and discussed until consensus was reached. The other assessors were involved in this process if needed. Full texts were then imported into EndNote for all citations that appeared to meet the inclusion criteria. Four of the reviewers (C. F., E. F., S. W., J. Ö.) then reviewed all full texts (Figure 1). Disagreements in full‐text reading were resolved as previously stated. Quality appraisal of the eligible full‐text reviews was independently performed by two assessors (V. A., N. L.), according to the JBI critical appraisal checklist for systematic reviews and research syntheses.^16^ Disagreements between assessors were resolved as previously stated (see Appendix B). No reviews were excluded based on the quality appraisal. A total of 31 records were included in the review (see Table 1). Reasons for exclusion of full‐texts are summarized according to PRISMA 2020 flow diagram^19^ presented in Figure 1.

**Table 1:** Included 31 reviews

Author (year)	Term/setting	Focus of review	Review type/number of included studies	Time frame of included studies	Method of synthesis	Outcome measures used in included studies	JBI Score (max: 11)
Dall'Oglio et al. (2018)	Family‐centred care/neonatal ICU	Properties and quality of instruments to measure parent's satisfaction with FCC	Systematic review/*n* = 11	2006–2015	Synthesis guided by a data extraction form created by the authors of the review	Self‐reported through validated questionnaires	9
Brouwers et al. (2017)	Patient centredness, patient‐centred communication/not specified	Quality of 13 instruments designed to measure PCC in patient–doctor communication	Systematic review/*n* = 13	1991–2013	Methodological quality of the instruments measured with COSMIN checklist	Not applicable	10
Coyne et al. (2018)	Family‐centred care, person‐centred care and child‐centred care/not specified	Differences, meanings and similarities of concepts of centeredness, i.e., FCC, PCC and CCC	Concept analysis/*n* = 35	2012–2016	Rodger and Knafls (2000) method for concept analysis	Not applicable	8
Maassen et al. (2017)	Patient‐centred care/outpatient psychiatric services	Patient's perspectives of ‘good care’ as compared to academic perspectives of PCC	Narrative literature review/*n* = 12	2000–2014	Literature review complemented by qualitative exploratory research to compare the perspectives	Patient perspectives were collected through focus group discussions and interviews	8
De Kok et al. (2018)	Patient centredness/high income settings	Role of provider–client relationships in adherence to ART	Critical review/*n* = 44	Studies published after 1997	An aggregative review combined with an interpretative review	Observational and self‐reported	9
Chiang et al. (2018)	Patient‐centred care/not specified	The effect of nurse‐led PCC. Outcomes included behavioural risks (smoking, physical activity) and physiological parameters (body weight, blood pressure, blood glucose, blood lipoproteins), health‐related quality of life, mortality and self efficacy	Systematic review/*n* = 15	1988–2017	Meta‐analysis	No specific methods of outcome assessments	11
Kim and Park (2017)	Person‐centred care/long‐term care facilities and home settings	Effectiveness of PCC on dementia patients, outcomes include depression, agitation, QoL and NPS	A systematic literature review/*n* = 19	1998–2016	Meta‐analysis	Assessed by carers through different scales and instruments, i.e., Cornell Scale for Depression in Dementia, Neuropsychiatric Inventory–Nursing Home	11
Brooke and Ojo (2018)	Person‐centred care/acute hospitals	Elements of a ‘sustainable, competent and empathetic workforce’ presented through various themes, including *understanding the current workforce*, *implementation and evaluation of training* and *exploration of new and existing roles*	Literature review/*n* = 12	2006–2016	Meta‐synthesis	Outcomes for dementia patients were mainly observational (assessed by carers) Perspectives of carers were self‐reported through various means (questionnaires, interviews)	8
Larsen et al. (2018)	Client‐centred practice/not specified	In what ways the COPM could enhance CCP, i.e., conditions required for, and how to use the COPM for enhancement of CCP	Scoping review/*n* = 12	2005–2016	Qualitative content analysis	Wide variety of methods used (Observational, questionnaires, interviews and focus groups) with both OTs and patients	8
Lloyd et al. (2018)	Person centeredness/any rehabilitation setting	Recommendations for practice regarding goal setting within stroke rehabilitation	Systematic review/*n* = 4	2011–2016	Meta‐aggregation	Patient's experiences of stroke rehabilitation collected through various qualitative data collection methods (semi‐structured interviews only one specifically mentioned)	10
Gondek et al. (2017)	Patient‐centred care, person‐centred care/child or youth mental health setting	Facilitators and barriers of PCC in mental health services for children and young people	Systematic review/*n* = 23	2004–2015	Narrative synthesis (Popay et al., 2006)	Perspectives of professionals, service users and carers were collected mainly through interviews and focus groups	10
Rea et al. (2018)	Family‐centred rounds/paediatric care settings	Various factors of parents experiences of FCR (satisfaction, relationship with carers, knowledge of care etc)	Systematic review/*n* = *28*	2007–2017	Meta‐analysis	Variety of methods used, mainly surveys but also observations, interviews and instruments	8
Hill et al. (2018)	Family‐centred care/PICU	Parental experiences of FCC in PICU based on each IPFFC core concept	Integrative review/*n* = 49	2007–2016	Synthesis was guided by an extraction template structured to relate to each core concept of FCC	Parental experience were collected through interviews and surveys complemented with observations	10
McCalman et al. (2017)	Family‐centred care/primary healthcare services	Strategies for, and enablers of, FCC in primary healthcare services for indigenous people. Health outcomes for indigenous children and parents/caregivers	A systematic scoping review/*n* = 25	2000–2015	Grounded theory method	Wide variety of methods of assessment used. Qualitative methods included mainly interviews in lone or group settings	11
Okrainec et al. (2017)	Patient‐centred care/not specified	The impact of patient‐centred discharge tools. Outcomes included patient experience, satisfaction, mental status, self‐efficacy, knowledge and comprehension of care, adherence, mortality and unplanned visits/readmission/LOS	Systematic review/*n* = 30	1995–2014	Descriptive narrative synthesis	Not clear in the review	9
Archer and Meyer (2018)	Patient centredness/undergraduate medical curricula	Nine interventions that possibly could develop PCC for medical students such as reflection, small‐group discussions etc.	Scoping review/*n* = 58	2000–2017	Content analysis to produce categories	Not clear in the review	8
Diamond‐Smith et al. (2018)	Person‐centred care/family planning services	The impact of interventions to improve PCC quality of family planning services. ‘Clinical outcomes’ (decreased rates of unintended pregnancies, increased family planning uptake) and ‘person centred outcomes’ (experience of, satisfaction with care)	Narrative review/*n* = 25	1990–2014	Descriptive narrative synthesis	Outcomes exclusively focused on clients' perspectives. Not clear how data were collected (interview, survey etc.)	11
Du Toit et al. (2019)	Person‐centred care/residential dementia care	Two constructs for enhancement of meaningful engagement with patients suffering from dementia (*promoting a culture of collaborative care* and *understanding the resident as a person with a past, present and future*)	Critical interpretive synthesis/*n* = 26	1997–2016	Critical interpretative synthesis	Observational (assessed by carers through DCM, ATOSE etc.)	8
Goldfarb et al. (2017)	Patient‐centred care, family‐centred care/adult ICU	Outcomes of PCC and FCC interventions, such as morbidity, mortality, self‐reported satisfaction, psychologic symptoms, functional status, quality of life, use of life‐sustaining therapies, length of critical care unit or hospital stay and cost of care	Systematic review/*n* = 46	1995–2016	Meta‐analysis	Exclusively patient‐ and family outcomes. Self‐reported outcomes, such as satisfaction and anxiety, were assessed through different instruments (i.e., Family Satisfaction ICU, HADS and CCFNI)	10
Almasri et al. (2018)	Family‐centred care/various care settings for children with physical disabilities	Parents of children with physical disabilities perception of to what extent the care their children received was family centred	Systematic review/*n* = 15	2004–2017	Meta‐analysis	Parental experiences were self‐reported and assessed through MPOC‐20	10
Lloyd et al. (2018)	Person‐centred coordinated care/not specified	PRMs that can be used to measure factors of P3C were mapped against a theoretical model of P3C and used to create a web‐compendium of PRMs	A pragmatic approach for the identification of P3C‐PRM/*n* = 63	2014–2016	P3C‐PRMs were identified and published in online compendium. These were later mapped against a theoretical model of P3C to shortlist generic measures of P3C	Not applicable	8
Lepore et al. (2018)	Person‐directed care planning/mainly nursing homes but PDCP processes in any care setting were included	Five different themes of PDCP (facilitators, barriers, the concept of PDCP, essential elements of PDCP and outcomes of PDCP)	Scoping review/*n* = 64	2006–2015	Thematic content analysis	Outcomes were measured for both carers and patients in the included studies. Factors included Patient engagement, asking patients about their preferences; however, it is not clear in what ways these were measured	8
O'Loughlin et al. (2017)	Patient‐centred/patient‐ centred medical homes	Different factors of patient‐reported experiences within PCMH (access to care, patient–physician and patient–practice relationships, patient engagement, goal‐setting etc)	Scoping review/*n* = 39	2007–2016	A thematic approach was used to describe patient‐reported experiences within PCMH	Wide variety of methods used to assess self‐reported patient experiences (Survey‐tools, various forms of interviews, focus groups etc.)	7
Menczykowski et al. (2018)	Family‐centred care/home setting	Various factors of FCC early discharge programmes, such as components of and readmission rates to the programme and time of transition to full oral feeding. These were complemented by both physiological outcomes (infant weight gain, breastfeeding data) and psychological outcomes (parent's experiences)	Systematic review/*n* = 8	2009–2016	Evidence synthesis	Outcomes were both observational and self‐reported. Self‐reported outcomes were collected through different means (interviews, focus groups, surveys etc.)	10
Ludlow et al. (2018)	Person‐centred care/residential aged care	Various dimensions of hearing loss and its effects on achieving PCC (communication breakdown, social isolation and reduced social participation, limited access to hearing services, inadequate training provided to care staff etc.)	A two‐stage narrative review/*n* = 6	2004–2014	General inductive analysis	Outcomes were both self‐assessed and observational. Patients reported through various means, including questionnaires, interviews and surveys	9
Mackie et al. (2018)	Patient‐ and family‐centred care (PFCC)/adult acute care wards	Components and outcomes of interventions that promote family involvement in care, such as strategies used to implement the interventions and patient outcomes	Integrative review/*n* = 11	2003–2014	The synthesis was guided by the method described by Whittemore and Knafl (2005)	Patient outcomes were both observational and self‐reported; however, it is not clear through what means this was assessed	10
Brooke et al. (2018)	Person‐centred care/various care settings for patients with dementia	Four themes regarding healthcare professionals and care workers culture and its impact on their ability to provide PCC	Systematic review/*n* = 7	2011–2016	Meta‐synthesis	Outcomes focused on healthcare professionals and care workers	9
						Both observational and self‐assessed through various means	
Arakelian et al. (2017)	Person‐centred care/perioperative nursing	Four themes regarding patient's perspectives of the meaning of PCC	Integrative review/*n* = 23	1990–2013	The synthesis was guided by the method described by Whittemore and Knafl (2005) and Friberg et al. (2012)	Exclusively self‐reported outcomes were collected through various means (interviews, surveys, questionnaires)	9
Allen et al. (2018)	PFCC/chronic disease settings	Three elements of PFCC were identified. The outcomes were: *patients and practitioners felt able to engage with each other on an emotional and social level, patients and families felt empowered to be part of the care process* and *patients and families experienced care as effective at addressing their individual needs*	Systematic review/*n* = 24	1999–2015	Qualitative meta‐synthesis	Outcomes focused on patients and their family members and were exclusively self‐reported. Data were collected mainly through interviews and focus groups. One included study performed a literature analysis	9
Carruthers et al. (2018)	Patient‐centred care/ICU	Experiences of mechanical ventilation survivors presented in three themes complemented with in what way healthcare professionals could help facilitate PCC	Qualitative meta‐ethnography/*n* = 38	1994–2017	Meta‐ethnography and qualitative synthesis	Outcomes were both self‐reported and observational	10
Poitras et al. (2018)	Patient‐centred care/not specified	Elements of seven different PCC interventions with positive health‐related outcomes presented in three themes: *patient‐oriented interventions, professional interventions* and *organizational interventions*	Scoping review/*n* = 52	2012–2016 (reviews), 1990–2015 (original articles)	Inductive analysis	Outcomes were health‐related and focused on patients with multimorbidty	7

Abbreviations: ART, antiretroviral therapy; ATOSE, Assessment Tool for Occupational and Social engagement; CCC, child‐centred care; CCFNI, Critical Care Family Needs Inventory; COPM, Canadian occupational performance measure; COSMIN, consensus‐based standards for the selection of health measurement instruments; DCM, dementia care mapping; FCC, family‐centred care; FCR, family‐centred rounds; HADS, Hospital Anxiety and Depression Scale; ICU, intensive care unit; IPFFC, Institute for Patient and Family‐Centred Care; JBI, Joanna Briggs Institute; JBI SCORE, JBI Critical appraisal checklist score; LOS, length of stay; NPS, neuropsychiatric symptoms; OT, occupational therapist; P3C, patient‐centred coordinated care; P3C‐PRM, patient‐centred coordinated care patient‐reported measures; PCC, patient‐centred care; PDCP, patient‐directed care planning; PICU, paediatric intensive care unit; PRM, patient‐reported measures; QoL, quality of life.

### 2.4 Data charting process

A preliminary data charting form was developed and tested by four members of the team (C. F., E. F., S. W., V. A.) and then discussed with the remaining members. Data charting was subsequently conducted in NVivo by one reviewer (V. A.) in continuous discussion with the team. Data on citation characteristics, such as year of publication, type of review, centredness term used and results connected to centeredness were abstracted. In the included records, the terms used together with ‘centredness’ were person/patient/family/child and client (see Table 1).

### 2.5 Method of synthesis

The abstracted data connected to centredness in health care included text segments from the result sections and were analysed by thematic analysis informed by Braun and Clarke^20^ and the description of analyses of reviews by Finfgeld‐Connett.^21^ First, the review results were read several times to get a naïve understanding of potential themes. A theme represents a patterned response or meaning within the data set and captures something important about the data in relation to the research question.^20^ Text segments with similar components were grouped into potential themes and subthemes. The analysis continuously moved back and forth between the entire data set, the abstracted text segments, and the forming and refinement of conceptual themes. Our analysis evolved into data being sorted into three main themes: *Attributes of centredness*, *Translation of theory into practice* and *Evaluation of effects*. Further identification of subthemes (for each main theme) was also done.

## 3 RESULTS

The included 31 reviews encompassed a total of 837 original studies published from 1988 to 2017. The majority of these studies were carried out in western countries (North America 45%, Europe 32%, Australia and New Zealand 11%), while 8% were carried out in Asia and Middle East, 3% in Africa and 1% in South America. The terms used in the reviews were patient‐centred (36%), person‐centred (33%), family‐centred (25%), client‐centred (3%) and child‐centred (3%). The 31 reviews focused mainly on clinical settings; 11 in inpatient hospital settings, including intensive care units and 14 reviews in outpatient settings, including primary care, long‐term care, home care and rehabilitation. The remaining reviews included one review covering medical education, two reviews on measures to evaluate centredness in health care and one review studying concepts. The populations targeted in the reviews were patients (53%), parents or family members (32%) or staff (15%).

**Figure 2:**
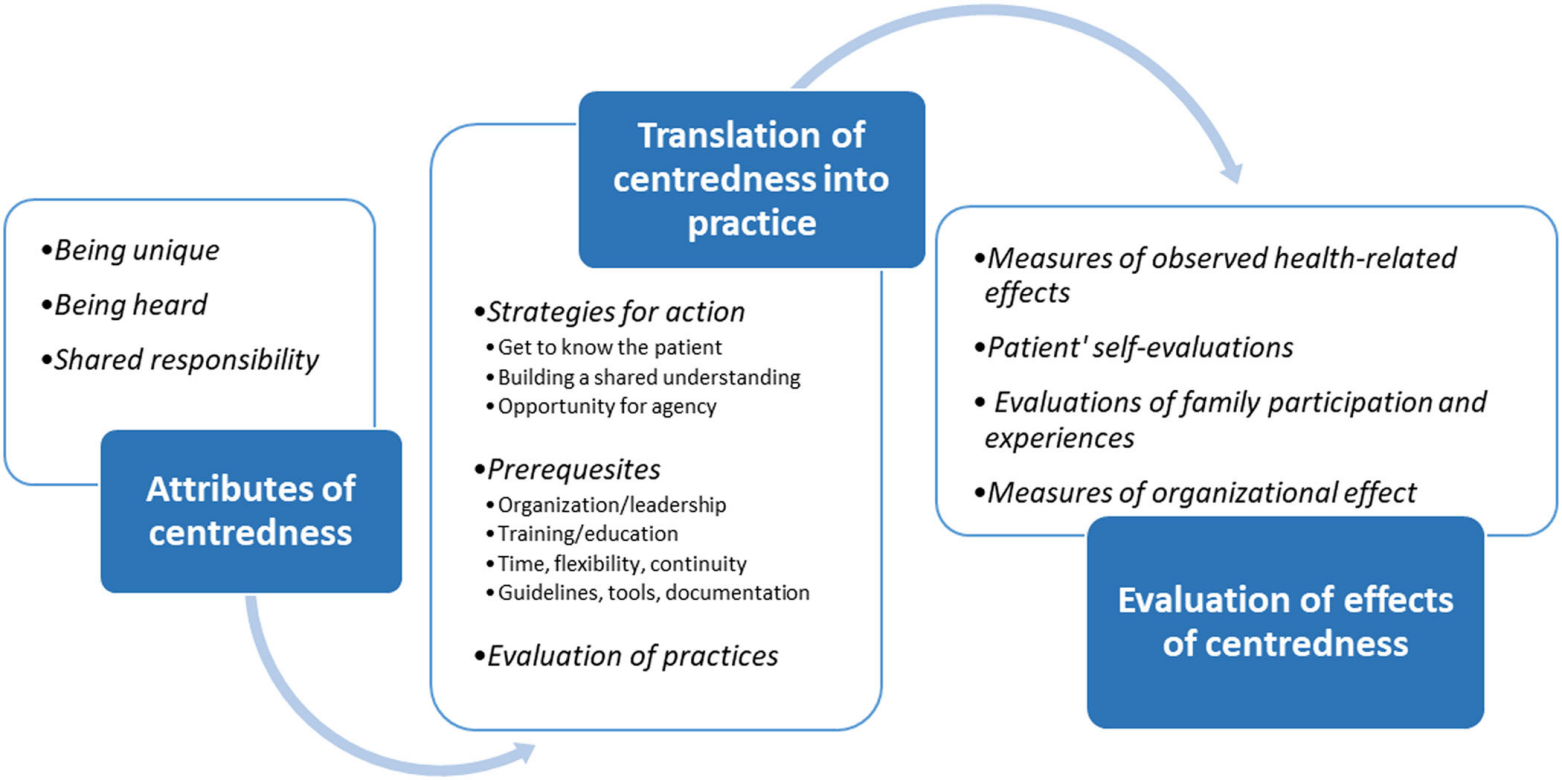
The three main themes and their relation to each other in a care process

Three main themes were identified in the synthesis of reviews, which relate to each other in a care process, reflecting centredness in health care: *Attributes of centredness, Translation from theory into practice and Evaluation of effects of centredness*. The first theme ‘Attributes of centredness’ describes the essence of what centredness in health care encompasses. Further, ‘Translation of theory into practice’ describes how centredness is carried out in practice, while ‘Evaluation of effects’ describes possible measures and effects of practising centredness in health care (see Figure 2).

### 3.1 Attributes of centredness

Four reviews elaborated explicitly on theoretical/philosophical/ethical aspects of centredness in health care. The reviews described patient and family experiences of loss of autonomy when suffering from illness and disease, giving rise to feelings of helplessness and dependency on others. When interacting with health care, the patients felt it was important their concerns and suffering were taken seriously. Patients expressed a sense of being forced to rely on health care to protect their life and body, and felt it was important that practitioners showed consideration, tact and discretion in caring for them and recognized the patients' ownership of their bodies and power in their own lives.^22^, ^23^, ^24^, ^25^

According to these reviews, centredness comprised the attributes: *being unique, being heard* and *shared responsibility*.

#### 3.1.1 Being unique

This first attribute includes being treated as a human being with basic rights and being treated as a person with a unique personality and life.^22^, ^23^, ^24^, ^25^ A person is an experiencing individual, with their own way of perceiving and experiencing and with a heterogeneous response to illness.^22^ This attribute is about acknowledging that the person, who in this particular situation is a patient, is much more than their diagnosis.^22^, ^24^ Being a person entails both body and soul, with a focus on strengths and competencies, but also vulnerabilities. Being unique embraces the fact that the experience of illness and symptoms differs between people and has different levels of impact on their daily life.^22^, ^23^, ^24^

#### 3.1.2 Being heard

The second attribute entails that every patient should be allowed to share their story^24^ and has the right to be heard.^22^ When a person shares their story, their preferences, needs and values, as well as perceptions and experiences, are usually articulated. Being heard embraces autonomy, privacy and integrity, as well as dignity and respect.^22^ Sharing experiential knowledge supports the patient in being appreciated as a person with knowledge, and acknowledged as an expert who can contribute to the care process.^22^, ^23^, ^24^ However, experiences, perceptions and desires are not stable. Instead, they are situational and may change over time, leading to changes in understanding, ability and needs.^22^, ^23^

#### 3.1.3 Shared responsibility

The last attribute highlights the patient's desire and right to participate in their own care. The reviews describe mutual participation, including patients, significant others and healthcare professionals sharing power and responsibility.^22^, ^23^, ^24^, ^25^ This shared responsibility is described as a joint venture in which the healthcare professional, as medical expert, holds a professional responsibility, while the patient's responsibility is based on ability and wish.^22^, ^23^ Shared responsibility is founded on trust and safety.^22^, ^23^, ^25^ Patients have a right to take part in choices and decisions about their care.^22^, ^23^, ^24^

### 3.2 Translation of theory into practice

Twenty‐one reviews elaborated on how concepts of centredness are translated into practice. These reviews underlined that the essence of centredness is expressed through action and emerges in the interaction between patient, family and health care providers. Guidelines, aspirations, and theory of ‘centredness’ may help but do not necessarily translate into practice.^26^ The interactional practices of centredness require the health care providers to have communicative skills and a flexible approach, as each person has different preferences and capabilities. In addition, quite a few prerequisites must be fulfilled to facilitate the translation of centredness theory into practical care.

#### 3.2.1 Strategies for action

The concept of centredness in health care is described as a practice that manifests itself in the interaction with the patient. Health care providers are responsible for facilitating this interaction, which requires sensitivity and attentiveness. Language, communicative difficulties and hearing loss, as well as culture, are factors that need to be taken into account and are described as barriers to interaction.^27^, ^28^, ^29^ Strategies to enable interaction included *Getting to know the patient*, *Building a shared understanding* and *Enabling opportunity for agency*.

It is important to *get to know the patient*, who the person is^26^, ^30^, ^31^; that is, what matters to the person, what their illness experience is and their needs and preferences. Getting to know someone may include listening to a personal narrative concerning things that happened in the person's past that may affect who they are now or with whom the person has important relationships.^30^, ^31^ As a healthcare practitioner, you can either encourage people or deflate them, leaving them feeling empowered or disempowered.^26^ Several reviews describe the importance of listening and building trust by being present and attentive.^26^, ^30^, ^32^

*Building a shared understanding* by sharing information and exchanging knowledge between patient, significant others and professionals are seen as important in finding a common ground. A patient suffering from illness and disease might feel a loss of autonomy and a dependency on others,^22^ but patients are still crucial actors in their own care. Patients' specific knowledge concerns their experiential knowledge of their health condition, illness experience, goals and life context.^30^, ^31^, ^33^, ^34^, ^35^ Information from health care aims to support people in making informed decisions about their health and care, and adapting to each patient's specific needs, capabilities, resources and circumstances.^30^, ^35^ In interviews, patients emphasize the importance of healthcare professionals being knowledgeable and competent, although, at the same time, the medical authority may cause uncertainty and hinder participation.^30^, ^31^, ^33^, ^34^, ^35^ This implies a balance between active and open listening and providing professional knowledge and expertize.^22^ Information videos, leaflets or educational programmes may improve communication and contribute to building a shared understanding.^36^

*Enabling opportunity for an agency* is the third strategy for action, implying welcoming the patient as part of the team and enabling opportunity for the patient and/or significant others to act.^30^, ^31^, ^32^, ^33^, ^37^, ^38^ However, healthcare professionals must be sensitive to the extent to which the patient and significant others wish to engage in care planning and decision‐making,^26^, ^31^, ^32^ as not everyone wishes to be involved. How much patients and significant others wish to be involved depends on connection, trust and their sense of ease, which may change over time.^26^, ^30^, ^32^, ^33^

#### 3.2.2 Prerequisites

The reviews describe many prerequisites to facilitating practices of centredness in healthcare, including *The organization/leadership*, *Training and education of healthcare staff*, *Time, flexibility and continuity*, and *Guidelines, tools and documentation*.

*The organization/leadership* plays a major role in setting the agenda, forming a vision and allocating resources. To implement practices of centredness, a supportive organizational structure is necessary, creating a collaborative environment, consistency, effective partnerships with other services and providing necessary means.^37^, ^39^ Necessary means refers to both material and contextual means, as well as support and empowerment to be able to respond to patient needs and bend the rules if necessary. Healthcare professionals treated as a complete person by the organization will reciprocate with a practice centred on the patient in return.^37^ Practising centredness is not only about the person behind the patient but also about the person behind the health professional.^27^, ^35^, ^37^, ^40^ Lack of influence over policies, procedures and practices creates feelings of disempowerment among healthcare professionals.^27^ Along with an increasing workload and a lack of support, this negatively affects their psychological well‐being and capability to practice centredness in health care.^27^, ^37^, ^40^

*Staff training and education* were described in the literature as the most important aspect for the successful implementation of centredness in health care. It is not enough to have a kind and well‐meaning workforce—skill and training are required. However, the content of this training and education was scantily described. The few reviews^27^, ^35^, ^37^, ^38^, ^41^, ^42^ providing descriptions stated that the training and education focused on the theory of centredness and the practice of centred health care, including empathy and compassion, communication and interactional skills, and shared decision‐making. Cultural sensitivity training was also raised.^40^ Central components of training were reflection, discussion and feedback.^35^, ^42^ Role‐play was described as one strategy, performed by students, which could prepare healthcare staff for complex situations, leading to increased self‐confidence in practicing centredness in health care.^42^

*Time, flexibility and continuity* were attributes described as facilitating practice. Time is important in order to listen and build relationships.^37^, ^39^ Stressed or busy healthcare professionals were described as an obstacle to interaction with patients,^26^, ^33^ while flexibility within the services, policies, procedures and practices (e.g., flexible visiting hours) facilitated the interaction.^33^, ^36^, ^37^, ^39^, ^40^, ^41^ Consistency of staff was described as an important facilitator for practicing centredness, as the relationship between staff and patients is founded on trust.^33^, ^39^, ^40^

*Guidelines, tools and documentation of care plans* were ways to facilitate centredness in health care.^30^, ^31^, ^35^, ^36^, ^38^, ^39^, ^41^, ^43^, ^44^ For example, an interview tool (Canadian occupational performance measure)^30^ was used to increase awareness of the patients' needs, preferences and values, as well as enable the processes of forming partnerships and developing collaborative goals. The use of protocols was found to help healthcare providers plan and coordinate care and to create structured care plans that reflect patients' needs, specific conditions, personal challenges and goals.^38^ The care plans may also include the needs of family and significant others.^38^

#### 3.2.3 Evaluation of practices of centredness

Four reviews examined a total of 80 tools and instruments to assess experiences of centredness in practice and the degree to which it is applied, that is, tools to safeguard practices. The majority of the instruments were patient‐reported evaluations to capture patients' perspectives on aspects of centredness (*n* = 74).^45^, ^46^ The assessment tools also included questionnaires reported by parents (*n* = 3),^47^, ^48^ and observation tools in which aspects of centredness (e.g., communication skills) were assessed by an outside observer (*n* = 3).^45^

The included reviews mapped patient‐reported evaluations for dimensions relating to a model for coordinated care,^46^ instruments for measuring parent satisfaction with neonatal intensive care units,^47^ the extent of centredness in health care^48^ and instruments developed for increasing centredness in patient‐professional communication.^45^

### 3.3 Evaluation of effects of centredness

Fourteen reviews contained results regarding measuring the effects of centred interventions or practices. The reporting of the effects in these reviews was diverse and no synthesis of effects could be outlined. The reported measures used to evaluate effects have been divided into four categories: (1) measures of observed health‐related effects; (2) patient‐reported evaluations; (3) evaluations of family participation and experiences and (4) measures of organizational effects

#### 3.3.1 Measures of observed health‐related effects

Nine reviews contained results from studies evaluating measures of the observed health‐related effects of centred care.

Two reviews registered mortality and measured health by body mass index, blood pressure, cholesterol levels and blood glucose.^36^, ^49^ Two reviews measured the health of infants and children by nutritional status, birth weight and weight at discharge for infants and weight for children aged 0–5.^40^, ^43^ Data on health habits were also collected, including smoking^49^ and adherence to diet, exercise and medication.^32^, ^50^ Further evaluation included observed symptoms of agitation,^44^ knowledge of family planning^35^ and patient activation and goal setting.^51^

#### 3.3.2 Patients' self‐evaluations

Seven reviews included studies of centred interventions that used patient' self‐evaluations of the interventions.

These evaluations included health and well‐being, such as quality of life,^31^, ^44^ health‐related quality of life dimensions, physical functioning, pain, anxiety, self‐efficacy and emotional role limitation.^41^, ^49^ In addition to assessing health and wellbeing, two reviews reported evaluations of feeling validated, comforted^31^ and feeling understood in their needs,^30^ as well as patients' understanding of their own issues and experiences^30^ and capability to make choices about their own care.^31^ Family‐centred interventions for indigenous children reported evaluations of the children's emotional and preventive health, along with separation distress and anxiety.^40^ Only one review regarding patient‐centred medical homes reported evaluations of patient satisfaction.^51^

#### 3.3.3 Evaluations of family participation and experiences

Five reviews reported results from interventions that had observed or reported evaluations by family members of the patient. These evaluations included satisfaction with and comprehension of care, understanding of information, communication and decision‐making, as well as stress and anxiety.^34^, ^36^, ^40^, ^43^, ^52^ Evaluations of parents of indigenous children included substance abuse, depression, anxiety, trauma and parenting and caregiving knowledge.^40^

#### 3.3.4 Measures of organizational effects

Seven reviews summarized the effects on healthcare organization.

The effects measured in the reviews were healthcare utilization,^40^, ^50^ consultations and opportunity for follow‐up,^40^ length of stay,^36^, ^41^ costs^36^, ^40^ and quality and safety.^31^ Other measured effects were access to care^51^ and readmission rates.^43^, ^50^

## 4 DISCUSSION

Three main themes were identified in our synthesis: (1) important attributes of centredness, (2) how centredness in health care translates into clinical practice and (3) measures of the effects of practising centredness in clinical care. These identified themes tell us that the literature on centredness in health care is broad and involved both exploring what centredness encompasses and how it can be enacted, as well as exploring how it can be measured and safeguarded. Concerning what centredness encompasses, our results highlight three important common attributes that indicate theoretical underpinnings of an existential philosophical character: being treated as unique, being heard and shared responsibility. However, these findings were synthesized from four reviews that mainly focused on the views of patients as to what constitutes centredness, or in one review, what constitutes ‘good care’.^22^, ^23^, ^24^, ^25^ Our three aspects are included in a previous presentation on ten common important aspects of centredness by Hughes et al.,^13^ and in the nine themes presented by Håkansson and Eklund.^12^ These presentations suggest an overlap among types of centredness^12^, ^13^ and that the concept of centredness is antiessentialist and a multidimensional woven fabric of fibres.^13^ The reviews included in this overview used the terms patient‐, person‐, family‐, child‐ and client‐ in combination with the concept of centredness (see Table 1). More importantly than the term used is the understanding of the concept in the context of the practices in which they have been used.^13^ As this first theme in our synthesis is based on four reviews that mainly explore patient views, one can nevertheless wonder whether the three attributes found in our study are perhaps of particular importance to patients. The more extensive collection from, for example, Hughes et al.^13^ is built on both professional and patient perspectives.

The complexity in actually practising centredness is visualized in the second theme identified in our synthesis, which can be said to be more of a reflection of the professional view. Here we highlight that the core of centredness is created in actions and in communication between patient, family and health care providers. Getting to know the patient, finding a common ground and opportunity for agency/taking part are crucial aspects in this process. Our results also identify prerequisites for this interaction to take place, that is, management, staff training and education, time, flexibility, continuity, guidelines, tools and documentation. The multifaceted complexity involved in translating theory into practice is also discussed by, for example, Sharma et al.,^14^ McCormack et al.^53^ and Anell and Nolte.^54^ As in our study, they highlight that centred care is an act based on relationship and communication.^14^, ^53^ Practising centredness includes removing obstacles to patient involvement and empowering the patient so they can take part in decisions.^14^, ^53^, ^54^

Regarding the evaluation of the effects of centredness, no synthesis of effects could be outlined due to the diverse outcomes reported in the included reviews. Furthermore, synthesis would be outside the scope of this study, which overviewed reviews published during a limited period. Instead, our synthesis focused on the measures used to evaluate effects and revealed several overarching themes as to which effects of centredness in health care are presented and which accompanying measures are used. The results show that the effect of interventions is often evaluated with medical measures defined by health care professionals rather than patients. This seems problematic when evaluating care that emanates from the patient's narrative and life context. Some reviews did include patients' self‐evaluations, including symptom experience and quality of life. However, whether patients actually desired to improve these outcomes was not generally discussed. Patient involvement in research is important for the development of valid instruments the patients find useful.^55^ Other forms of measures were effects on the health care organization and experience of family members. According to Sharma et al.,^14^ one main purpose of centred models of care is ‘satisfaction with care’. Only one review reported a measure of patient‐reported satisfaction with care and five on family‐reported satisfaction with care. Further, patient needs, preferences and wishes were described as important aspects of centredness, but also only reported in two reviews. It is remarkable that interventions that stress the involvement of patients do not ensure evaluations of what the patients find important are included. However, most reviews (11 out of 14) included both qualitative, quantitative and mixed‐method studies, with the presentation of specific measures given secondary importance. No distinctions regarding primary or secondary outcomes were reported in the reviews.

### 4.1 Method discussion, and limitations

To tackle the challenge of the considerable volume of publications on centredness in health care we decided to undertake an overview of reviews. We used an inclusive search strategy, encompassing a variety of search terms, which led us to focus on a select time frame for reviews to make the project feasible. However, our sample of reviews includes studies published between 1988 and 2017. Despite the inclusive search strategy this overview of reviews only covered a selection of the plethora of terms for centredness and centred care, and may therefore be seen as a ‘snap shot’ with no claim to provide an overall picture of centredness in health care. We also chose to focus on commonalities across concepts. This decision was informed by the result of an earlier review of reviews highlighting a number of similarities across the concepts of person‐centred and patient‐centred care.^12^ However, the focus on commonalities risks missing distinctions and differences across concepts. Nonetheless, the broadness of the field was clear and this overview of reviews has made it possible to relate important attributes of centredness (that are often treated separately in theoretical work) to clinical practice and evaluation. Our results point to opportunities to theorize on centredness in health care on an empirical basis, which may be meaningful in bridging an assumed theory‐practice gap. However, the results may only be seen as an indicative cross‐sectional snap‐shot, pinpointing common core denominators of centredness in health care.

To select relevant reviews we started from a normative base by broadly defining centredness in health care as framed by the University of Gothenburg Centre for Person‐centred Care and the ethics described in a position paper by Ekman et al.^17^ When selecting relevant reviews, the decision to start from a normative base may risk ending up in a circular argument of already having defined what we are searching for and what centredness in health care ought to be. Due to the inclusive search strategy, which resulted in a large number of reviews to be screened, the use of clear conceptualization was necessary.

To handle a large amount of literature, several reviewers took part in the screening process and this may affect the interrater reliability. However, all screening was performed in pairs and discussed until consensus was met; ambiguities within the pairs were discussed in the whole group.

## 5 CONCLUSIONS AND IMPLICATIONS

Our overview of reviews demonstrates that current synthesized research literature on centredness in health care is broad, as it focuses both on explorations of the conceptual basis and the practice, as well as measures of effects. The synthesis highlights commonalities related to centredness in health care of importance in the overall process ranging from theory to practice and evaluation, regardless of theoretical basis or terms used. The attributes identified point to the existential philosophical character of the field, and how it may be followed by translation to health care practice, which clearly implies or refers back to core attributes. However, the broad scope of outcomes identified was less clearly related to the attributes or the translation to practice and especially lacks clarification as to what extent the measures of effects reflect patient or other user priorities.

To further the understanding of the field of centredness in health care, a scoping review that more comprehensively maps the literature available is currently being undertaken by our team.

## CONFLICTS OF INTEREST

The authors declare no conflicts of interest.

## AUTHOR CONTRIBUTIONS

The study design and the underlying search strategy were developed by Caroline Feldthusen, Emma Forsgren, Sara Wallström, Joakim Öhlén, Eva J. Ung and Richard Sawatzky. Screening of records and reports were conducted by Caroline Feldthusen, Emma Forsgren, Sara Wallström, Joakim Öhlén and Eva J. Ung. Viktor Andersson and Noah Löfqvist performed the critical appraisal. Viktor Andersson conducted the data charting and Viktor Andersson, Caroline Feldthusen, Sara Wallström, Emma Forsgren, Noah Löfqvist, Eva J. Ung and Joakim Öhlén were involved in the analysis. The first draft of the manuscript was written by Caroline Feldthusen, Emma Forsgren and Sara Wallström. All authors were responsible for critical revision and finalizing of the manuscript. All authors read and approved the final manuscript.

## Data Availability

The data supporting the findings of this study are available from the corresponding author upon reasonable request.

## APPENDIX A

SEARCH IN PubMed

#1 (‘Person centered’[tiab]) OR (‘person centred’[tiab]) OR (‘person‐centered’[tiab]) OR (‘person‐centred’[tiab]) OR (‘person centeredness’[tiab]) OR (‘personcenteredness’[tiab]) OR (‘person centredness’[tiab]) OR (‘client‐centered’[tiab]) OR (‘client‐centred’[tiab]) OR (‘client centered’[tiab]) OR (‘client centred’[tiab]) OR (‘patient centered’[tiab]) OR (‘patient centred’[tiab]) OR (‘patient‐centered’[tiab]) OR (‘patient‐centred’[tiab]) OR (‘Patient‐Centered Care’[tiab]) OR (‘relationship‐centered’[tiab]) OR (‘relationship‐centred’[tiab]) OR (‘women‐centered’[tiab]) OR (‘women‐centred’[tiab]) OR (‘woman‐centered’[tiab]) OR (‘woman‐centred’[tiab]) OR (‘family‐centered’[tiab]) OR (‘family‐centred’[tiab]) OR (‘child‐centered’[tiab]) OR (‘child‐centred’[tiab])

AND

#2 (‘meta‐analysis’[Publication Type]) OR (‘meta analysis’[tiab]) OR OR (review[Publication Type]) OR (search*[tiab]) OR (‘critical interpretive synthesis’[tiab]) OR (‘integrative review’[tiab]) OR (‘narrative synthesis’[tiab]) OR (‘realist review’[tiab]) OR (‘meta‐ethnography’[tiab]) OR (‘meta interpretation’[tiab]) OR (‘meta‐summary’[tiab]) OR (‘meta‐study’[tiab]) OR (‘meta‐synthesis’[tiab]) OR (‘mixed‐studies review’[tiab]) OR (‘meta‐narrative review’[tiab]) OR (‘concept‐synthesis’[tiab])

## APPENDIX B

See Table B1

**Table B1:** Checklist for systematic reviews and research syntheses

Author	Q1	Q2	Q3	Q4	Q5	Q6	Q7	Q8	Q9	Q10	Q11	Score
Dall'Oglio et al.	Y	Y	Y	Y	N	N	Y	Y	N	Y	Y	8
Brouwers et al.	Y	Y	Y	Y	Y	Y	Y	Y	N	Y	Y	10
Coyne et al.	Y	Y	Y	Y	N	N	Y	Y	NA	Y	Y	8
Maassen et al.	Y	Y	Y	Y	N	N	Y	Y	NA	Y	Y	8
Kok et al.	Y	Y	Y	Y	NA	NA	Y	Y	N	Y	Y	8
Chiang et al.	Y	Y	Y	Y	Y	Y	Y	Y	Y	Y	Y	11
Kim and Park	Y	Y	Y	Y	Y	Y	Y	Y	Y	Y	Y	11
Brooke and Ojo	Y	Y	Y	Y	N	N	Y	Y	N	Y	Y	8
Larsen et al.	Y	Y	Y	Y	NA	NA	Y	Y	NA	Y	Y	8
Lloyd et al.	Y	Y	Y	Y	Y	Y	Y	Y	NA	Y	Y	10
Gondek et al.	Y	Y	Y	Y	Y	Y	Y	Y	NA	Y	Y	10
Rea et al.	Y	Y	Y	Y	Y	U	N	Y	NA	Y	Y	8
Hill et al.	Y	Y	Y	Y	Y	N	Y	Y	N	Y	Y	9
McCalman et al.	Y	Y	Y	Y	Y	Y	Y	Y	N	Y	Y	10
Okrainec et al.	Y	Y	Y	Y	N	N	Y	Y	Y	Y	Y	9
Archer and Meyer	Y	Y	Y	Y	NA	NA	Y	Y	NA	Y	Y	8
Smith et al.	Y	Y	Y	Y	Y	Y	Y	Y	N	Y	Y	10
Du Toit et al.	Y	Y	Y	Y	NA	NA	Y	Y	N	Y	Y	8
Goldfarb et al.	Y	Y	Y	Y	Y	U	Y	Y	Y	Y	Y	10
Almasri et al.	Y	Y	Y	Y	Y	Y	N	Y	Y	Y	Y	10
Lloyd et al.	Y	Y	Y	Y	NA	NA	Y	Y	N	Y	Y	8
Lepore et al.	Y	Y	Y	Y	NA	NA	Y	Y	NA	Y	Y	8
Loughlin et al.	Y	Y	Y	Y	NA	NA	Y	Y	N	N	Y	7
Menczykowski et al.	Y	Y	Y	Y	Y	U	Y	Y	Y	Y	Y	10
Ludlow et al.	Y	Y	Y	Y	Y	U	Y	Y	N	Y	Y	9
Mackie et al.	Y	Y	Y	Y	Y	Y	Y	Y	N	Y	Y	10
Brooke et al.	Y	Y	Y	Y	Y	U	Y	Y	NA	Y	Y	9
Arakelian et al.	Y	Y	Y	Y	Y	U	Y	Y	N	Y	N	8
Allen et al.	Y	Y	Y	Y	Y	N	Y	Y	NA	Y	Y	9
Carruthers et al.	Y	Y	Y	Y	Y	Y	Y	Y	NA	Y	Y	10
Poitras et al.	Y	Y	Y	N	N	N	Y	Y	N	Y	Y	7

Abbreviations: N, no; NA, not applicable; U, unclear; Y, yes.

*Source*: Joanna Briggs institute (2017).

JBI critical appraisal checklist for systematic reviews and research syntheses:

Q1: Is the review question clearly and explicitly stated?

Q2: Were the inclusion criteria appropriate for the review question?

Q3: Was the search strategy appropriate?

Q4: Were the sources and resources used to search for studies adequate?

Q5: Were the criteria for appraising studies appropriate?

Q6: Was critical appraisal conducted by two or more reviewers independently?

Q7: Were there methods to minimize errors in data extraction?

Q8: Were the methods used to combine studies appropriate?

Q9: Was the likelihood of publication bias assessed?

Q10: Were recommendations for policy and/or practice supported by the reported data?

Q11: Were the specific directives for new research appropriate?
